# Neuronal activity increases translocator protein (TSPO) levels

**DOI:** 10.1038/s41380-020-0745-1

**Published:** 2020-05-12

**Authors:** Tina Notter, Sina M. Schalbetter, Nicholas E. Clifton, Daniele Mattei, Juliet Richetto, Kerrie Thomas, Urs Meyer, Jeremy Hall

**Affiliations:** 1 Neuroscience and Mental Health Research Institute, Cardiff University, Cardiff, Wales UK; 2 Institute of Pharmacology and Toxicology, University of Zurich-Vetsuisse, Zurich, Switzerland; 3 MRC Centre for Neuropsychiatric Genetics and Genomics, Division of Psychological Medicine and Clinical Neurosciences, Cardiff University, Cardiff, UK; 4 Neuroscience Centre Zurich, University of Zurich and ETH Zurich, Zurich, Switzerland

**Keywords:** Neuroscience, Schizophrenia

## Abstract

The mitochondrial protein, translocator protein (TSPO), is a widely used biomarker of neuroinflammation, but its non-selective cellular expression pattern implies roles beyond inflammatory processes. In the present study, we investigated whether neuronal activity modifies TSPO levels in the adult central nervous system. First, we used single-cell RNA sequencing to generate a cellular landscape of basal TSPO gene expression in the hippocampus of adult (12 weeks old) C57BL6/N mice, followed by confocal laser scanning microscopy to verify TSPO protein in neuronal and non-neuronal cell populations. We then quantified TSPO mRNA and protein levels after stimulating neuronal activity with distinct stimuli, including designer receptors exclusively activated by designer drugs (DREADDs), exposure to a novel environment and acute treatment with the psychostimulant drug, amphetamine. Single-cell RNA sequencing demonstrated a non-selective and multi-cellular gene expression pattern of *TSPO* at basal conditions in the adult mouse hippocampus. Confocal laser scanning microscopy confirmed that TSPO protein is present in neuronal and non-neuronal (astrocytes, microglia, vascular endothelial cells) cells of cortical (medial prefrontal cortex) and subcortical (hippocampus) brain regions. Stimulating neuronal activity through chemogenetic (DREADDs), physiological (novel environment exposure) or psychopharmacological (amphetamine treatment) approaches led to consistent increases in TSPO gene and protein levels in neurons, but not in microglia or astrocytes. Taken together, our findings show that neuronal activity has the potential to modify TSPO levels in the adult central nervous system. These findings challenge the general assumption that altered TSPO expression or binding unequivocally mirrors ongoing neuroinflammation and emphasize the need to consider non-inflammatory interpretations in some physiological or pathological contexts.

## Introduction

The elucidation of neuroinflammatory mechanisms in psychiatric and neurological disorders is a highly active area of research that has the promise of establishing novel therapeutic strategies beyond classical nosologic boundaries [1–3]. Identifying and characterizing neuroinflammatory processes requires the availability of suitable and reliable biomarkers. Positron emission tomography (PET) using radiolabeled ligands selective for the 18-kDa translocator protein (TSPO) continues to be the most widely used technique to assess putative neuroinflammatory processes in-vivo [4–6]. TSPO is an evolutionarily conserved protein localized in the outer mitochondrial membrane, which has been linked to various (but not mutually exclusive) physiological processes, including cholesterol transport and steroid hormone synthesis, apoptosis and cell viability, redox processes and oxidative stress, and mitochondrial respiration and bioenergetics [4, 7]. Originally used to detect discrete neurotoxic changes in the central nervous system (CNS) [8], TSPO has commonly turned into a biomarker of “neuroinflammation” or even “microglial activation” [9–11]. By contrast, the possibility that TSPO levels could be altered by non-inflammatory stimuli is often overlooked, especially by researchers and clinicians concentrating on neuroinflammatory mechanisms in psychiatric and neurological disorders [4, 12]. This tendency may, however, introduce substantial interpretative bias in investigations using the TSPO biomarker. Indeed, whereas alterations in TSPO expression may have an inflammatory origin in some pathological contexts, it may as well mirror non-inflammatory processes in others [4].

Here, we examined neuronal TSPO levels under basal conditions and after neuronal activation in several mouse models. Whereas previous immunohistochemical studies identified TSPO protein in neuronal precursor cells and post-mitotic neurons [4, 13–15], it is currently unknown whether neuronal activity modulates TSPO expression in the brain. Based on the role of TSPO in mitochondrial bioenergetics [16, 17], we hypothesized that TSPO expression may mirror the increased demands in energy production under conditions of increased neuronal activity, and therefore, would be upregulated by the latter. To test this hypothesis, we first used single-cell RNA sequencing (scRNA-seq) to generate a cellular landscape of basal *TSPO* gene expression in the hippocampus of adult mice, whereas confocal laser scanning microscopy (CLSM) was used to verify TSPO protein in neuronal and non-neuronal cell populations. We then quantified TSPO mRNA and protein after stimulating neuronal activity with distinct stimuli, including designer receptors exclusively activated by designer drugs (DREADDs), exposure to a novel environment and acute treatment with the psychostimulant drug, amphetamine.

## Methods

### Animals

All experiments were performed using adult (12 weeks old) male C57Bl6/N mice (Charles Rivers, Sulzfeld, Germany). They were group-housed (4–5 animals per cage) in individually ventilated cages (Allentown Inc., Bussy-Saint-Georges, France) as described in detail elsewhere [18]. The cages were kept in a specific-pathogen-free (SPF) holding room, which was temperature- and humidity-controlled (21 ± 3 °C, 50 ± 10%) and kept under a reversed light–dark cycle (lights off: 09:00 AM–09.00 PM). All animals had *ad libitum* access to standard rodent chow (Kliba 3336, Kaiseraugst, Switzerland) and water throughout the entire study. All procedures were conducted during the dark cycle and had been previously approved by the Cantonal Veterinarian’s Office of Zurich. All efforts were made to minimize the number of animals used and their suffering. The number of animals in each experimental condition and series of experiment is summarized in Table S1 (Supplementary Information). The sample sizes in each group were estimated according to our previous studies exploring the cellular sources of altered TSPO expression in mouse models relevant to neuropsychiatric and neurodegenerative disorders [12].

### Single-cell RNA sequencing and analysis

Dissociation of hippocampal tissue and hippocampal cell isolation was carried out using mechanical dissociation at 4 °C as described in Supplementary Information. Left and right hippocampi from 3 animals were pooled for subsequent scRNA-seq analysis.

The quality and concentration of the single-cell preparations were evaluated using a haemocytometer in a Leica DM IL LED microscope and adjusted to 1000 cells/μl. 10,000 cells were loaded into the 10X Chromium controller and library preparation was performed according to the manufacturer’s indications (single cell 3′ v3 protocol). The resulting libraries were sequenced using the Illumina NovaSeq sequencer according to a depth of ~50,000 reads per cell. The scRNA-seq count matrix was analyzed using the Seurat (v3) R package [19, 20]. The data were filtered to include only cells from which expression was detected of more than 500 and fewer than 2500 unique genes, and from which fewer than 25% of the total counts were from mitochondrial genes. Read counts were log-normalized using a size factor of 10,000 molecules per cell and scaled by linear transformation. Cell clustering was performed using a dimensionality of 10 principle components and a resolution of 0.6. Cell-type assignments were predicted by matching representative expressed features to published cell markers data [21]. Specific gene sets for cell-type identification are provided in Fig. S1 (Supplementary Information). The scRNAseq raw data files have been deposited at the NCBI’s Gene Expression Omnibus [22] and are accessible through GEO Series accession number GSE143796. Functional network analyses were generated through the use of Ingenuity Pathway Analysis (IPA) (QIAGEN, Redwood City, CA, USA) as described before [23, 24].

### Quantitative real-time polymerase chain reaction (RT-PCR)

RNA extraction and quantitative RT-PCR analyses were performed according to previously established protocols [24, 25]. In brief, the animals were deeply anesthetized with an overdose of Nembutal (Abbott 23 Laboratories, North Chicago, IL, USA) and transcardially perfused with 20 ml ice-cold, artificial cerebrospinal fluid (CSF) (pH 7.4) [26]. After decapitation, the brains were immediately extracted from the skull, frozen on dry ice and stored at −80 °C until further processing. The brains were then cut into 1-mm coronal brain sections using razorblade cuts and subsequent micro-dissection of the medial prefrontal cortex (mPFC) (bregma: +2.0 to +1.5 mm), hippocampus (Hpc) (bregma −2.0 to −3.0 mm), nucleus accumbens (NAc) (bregma + 1.5 to +1.0 mm) and ventral midbrain (vMB) (bregma − 3.0 and −3.5). Total RNA was isolated using the RNeasy Plus Universal Mini Kit (Qiagen, Hilden, Germany). The procedure was conducted according to the manufacturer’s instructions, and the resulting RNA was quantified by the Agilent 2200 BioAnalyzer (Agilent Technologies, Santa Clara, USA). Quantitative RT-PCR was conducted with iScript one-step RT-PCR kits and a Taqman real-time system (CFX384, Bio-Rad Laboratories, Cressier, Switzerland) as previously described [24, 25]. A mouse TaqMan gene expression assay for *TSPO* (assay ID: Mm00437828_m1, catalogue number: 4331182; Thermo Fisher Scientific, Zurich, Switzerland) was used, whereas mRNA levels of *cFos*, *Arc* and *Zif268* were quantified using custom made primers (Table S2 in Supplementary Information). Ribosomal phosphoprotein (*36B4*; Table S2 in Supplementary Information) was used as the housekeeper control as validated previously [24, 25]. Relative gene expression was calculated with the 2^−ΔΔCt^ method [27]. All RT-PCRs and analyses were conducted by an experimenter blind to the experimental conditions. Blinding was performed in the form of coding by numbers.

### Immunohistochemistry and confocal microscopy

Immunofluorescence (IF) staining and CLSM were performed according to previously established protocols [12, 26]. In brief, the animals were perfused intracardially with oxygenated artificial CSF (pH 7.4), followed by 12 h post fixation in 4% phosphate-buffered paraformaldehyde (PFA) and cryoprotection [12, 26]. The brain samples were cut coronally with a sliding microtome at 30 μm (8 serial sections) and stored at −20 °C in cryoprotectant solution (50 mM sodium phosphate buffer (pH 7.4) containing 15% glucose and 30% ethylene glycol; Sigma-Aldrich, Switzerland) until further processing. For IF staining, the slices were rinsed with Tris buffer (pH 7.4) prior to incubation with the primary antibody. A summary of all antibodies used in this study is provided in Table S3 (Supplementary Information). The primary antibodies were diluted in Tris buffer containing 0.2% Triton X-100 and 2% normal serum, and the sections were incubated free-floating under constant agitation (100 rpm) overnight at 4 °C. The sections were then washed three times for 10 min in Tris buffer, incubated for 30 min at room temperature with secondary antibodies coupled to either Alexa488 (diluted 1:1000; Molecular Probes, Eugene, USA), Alexa568 or Alexa647 (each diluted 1:500; Jackson ImmunoResearch, Ely, UK) and DAPI (1 mg/mL H_2_O; Thermo Fisher Scientific, Zurich, Switzerland; diluted 1:20’000) Jackson ImmunoResearch, Ely, UK). After incubation, which was shielded from light, the sections were washed thoroughly three times for 10 min in Tris buffer, mounted onto gelatinized glass slides, coverslipped with Dako fluorescence mounting medium and stored in the dark at 4 °C. Data collection was performed with a confocal laser scanning microscope (LSM-700; Zeiss, Jena, Germany) as described in Supplementary Information. TSPO intensities co-localized with neurons, microglia, or astrocytes were measured and calculated using a custom macro (kindly provided by Prof. Jean-Marc Fritschy, Institute of Pharmacology and Toxicology, University of Zurich, Switzerland) for the ImageJ software. A detailed description of how TSPO co-localization was quantified is provided in Supplementary Information. All IF stainings and the corresponding analyses were performed by an experimenter blind to the experimental conditions. Blinding was performed in the form of coding by numbers.

### DREADD system

A DREADD-based chemogenetic approach was used to examine whether TSPO mRNA and protein levels change in response to selective neuronal activation. The following adeno-associated viruses (AAVs) were used: AAV8-hSyn1-hM3D(Gq)-mCherry (hM3D_Gq_V) and AAV8-hSyn1-EGFP (ConV). They were purchased from the Viral Vector Facility of the University of Zurich, Switzerland (www.vvf.uzh.ch) and were injected unilaterally in the medial prefrontal cortex (mPFC; anteroposterior [AP] = +2.0 mm, mediolateral [ML] = +0.3 mm, dorsoventral [DV] = − 2.0 mm, with reference to bregma) or the dorsal hippocampus (Hpc; AP = −2.0, ML = +1.6, DV = −1.9, with reference to bregma) using stereotaxic surgery. The latter was performed as described in Supplementary Information.

After a 2 weeks recovery period, the animals were injected with clozapine-N-oxide (CNO, 1 mg/kg; Enzo Life Sciences, Lausen, Switzerland) dissolved in 0.9% NaCl (B. Braun, Melsungen, Switzerland) or with 0.9% NaCl vehicle (Veh) solution only. The dose of CNO (1 mg/kg, i.p.) was chosen based on previous chemogenetic studies in rodents [28–30]. All substances were injected intraperitoneally (i.p.) using an injection volume of 5 ml/kg. After CNO or Veh injections, the animals were placed back into their home cages and killed 90 min (gene expression analyses) or 180 min (protein expression analyses) after injections as described above.

### Environmental and psychopharmacological manipulations

To study TSPO mRNA and protein levels following neuronal activation under physiological conditions, we compared animals exposed to a novel environment (NovE) and animals kept in their familiar home cage (HC). The NovE was a wall-enclosed open field arena (40 × 40 × 35 cm high) made of white polyvinyl chloride (PVC), which contained numerous unfamiliar objects (green LEGO^®^ DUPLO^®^ 2 × 4 brick, 64 × 32 × 24 mm^3^; black acrylic cylinder, 200 mm height, 50 mm diameter; transparent glass bottle, height 130 mm). In addition, the open field arena contained visual cues (black objects printed on white background), which were positioned on the arena’s inner walls. The animals were gently placed into the center of the arena and allowed to freely explore for 15 min, after which they were returned to their home cages before sacrificed 90 min (gene expression analyses) or 180 min (protein expression analyses) thereafter.

To study *TSPO* expression after psychopharmacological manipulations, we compared animals treated with saline (Sal; isotonic 0.9% NaCl) or d-amphetamine sulfate (Amph; Sigma-Aldrich, Switzerland) solution. Amph was dissolved in Sal solution and was administered at a dose of 2.5 mg/kg (i.p.) based on our previous studies [31]. All solutions were freshly prepared on the day of administration and were injected using an injection volume of 5 ml/kg. The animals were sacrificed for subsequent mRNA analyses (see above) 90 min after Sal or Amph treatment. The hypolocomotor response to the Amph challenge was confirmed by measuring the animals’ locomotor activity in an open field arena using the EthoVision (Noldus Technology, Wageningen, The Netherlands) tracking system as described before [31].

### Statistical analyses

A standard Seurat (v3) R workflow [20] was used for clustering and visualization of the scRNA-seq data, which included data scaling, principal component analysis and reduction, t-distributed stochastic neighbor embedding (tSNE), and uniform manifold approximation and projection (UMAP) clustering. Differential gene expression analysis in the scRNA-seq dataset was carried out using FindMarker functions, and *p*-values were calculated using Wilcoxon Rank Sum test with Bonferroni correction.

All RT-PCR and IF data met the assumptions of normal distribution and equality of variance. They were analyzed using independent Student’s *t*-test (two-tailed), whereas locomotor activity in the open field after Amph or Sal treatment was analyzed using a 2 × 18 (treatment × 5-min bins) repeated-measures analysis of variance (RM-ANOVA). These analyses were performed using SPSS Statistics (version 25.0, IBM, Armonk, NY, USA), with statistical significance set at *P* < 0.05 for all analyses. Exclusion of animals was not applied.

## Results

### Cellular landscape of basal *TSPO* expression in the mouse hippocampus

**Fig. 1:**
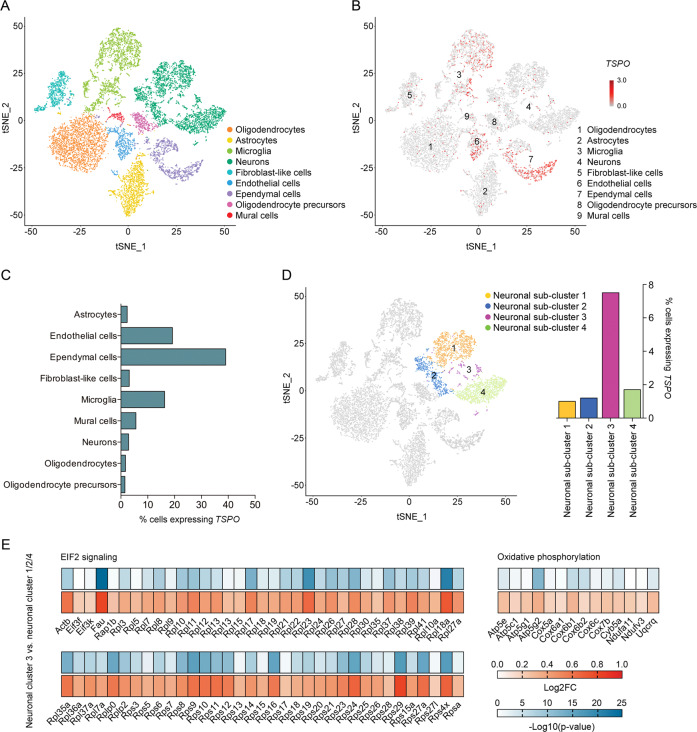
Cellular landscape of basal *TSPO* expression in the hippocampus of adult mice. **a** Visualization of single-cell RNA-sequencing (scRNA-seq) data using t-distributed stochastic neighbor embedding (tSNE), showing the clustering of 9 main cell populations. Cellular sub-clusters are provided in Fig. S3 (Supplementary Information). Corresponding uniform manifold approximation and projection (UMAP) scores are shown in Figure S2 (Supplementary Information). **b** *TSPO* expression (in red) in individual clusters of cells as detected by scRNA-seq. **c** Percentage of cells expressing *TSPO* in each of the 9 main cell populations, as measured by scRNA-seq. **d** tSNE scores of neuronal sub-clusters (neuronal clusters 1–4) and percentage of cells expressing *TSPO* in each of the 4 neuronal sub-clusters. **e** Differential gene expression (indexed as average log2 fold change, Log2FC) between neuronal cluster 3 and neuronal cluster 1/2/4, showing differentially expressed genes included in the eukaryotic initiation factor 2 (EIF2) signaling pathway and oxidative phosphorylation as identified by Ingenuity Pathway Analysis (IPA). The significance of differential gene expression is given as –Log10(*p*-value).

First, we obtained scRNA-seq of hippocampal cell suspensions from adult C57BL6/N mice in order to generate a landscape of basal *TSPO* mRNA expression in non-neuronal and neuronal cell populations. Clustering using specific gene-set enrichment for cell-type identification revealed 9 main cell populations (Fig. 1a; Fig. S2 in Supplementary Information), which could be broken down further into 18 clusters containing distinct cell sub-populations (Fig. S3 in Supplementary Information). As expected [12, 14, 15], basal *TSPO* mRNA expression was most abundant in ependymal cells, vascular endothelial cells and microglia (Fig. 1b, c). Detectable *TSPO* mRNA levels were, however, present in all other hippocampal cell populations as well, including neurons (Fig. 1b, c). Whilst ~3% of all neuronal cells were positive for *TSPO* mRNA (Fig. 1b, c), individual neuronal sub-populations showed a different *TSPO* mRNA expression profile (Fig. 1d). In three out of four neuronal sub-clusters (neuronal sub-cluster 1/2/4), *TSPO* mRNA was measurable in only 1.0 to 1.7% of cells, whereas *TSPO* mRNA was detected in 7.5% of cells in the remaining neuronal sub-cluster (neuronal sub-cluster 3) (Fig. 1d). Subsequent IPA of differentially expressed genes (neuronal sub-cluster 3 versus sub-clusters 1/2/4) demonstrated that the sub-cluster with higher *TSPO* mRNA expression (i.e., neuronal sub-cluster 3) was characterized by increased expression of genes involved in the eukaryotic initiation factor 2 (EIF2) signaling pathway (*z-*score = 6.16, −log(*p*-value) = 48.8; Fig. 1e). Consistent with the role of TSPO in mitochondrial bioenergetics [16, 17], neuronal sub-cluster 3 with high *TSPO* mRNA expression also showed high levels of gene expression pertaining to oxidative phosphorylation (*z*-score = 3.74, -log(*p*-value) = 4.8; Fig. 1e). Taken together, scRNA-seq confirmed the multi-cellular expression profile of TSPO under basal conditions and further showed that *TSPO* mRNA is detectable in various neuronal cell populations with a varying degree of basal gene expression.

**Fig. 2:**
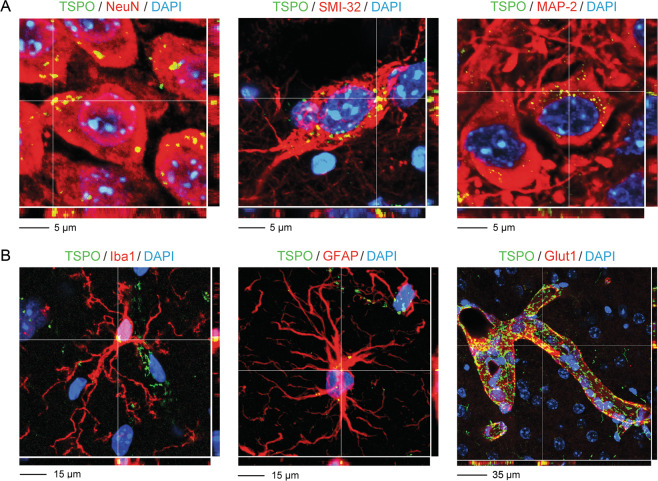
Immunohistochemical localization of TSPO protein in neuronal and non-neuronal hippocampal cells of adult mice using immunofluorescence staining analyzed by confocal laser scanning microscopy. The photomicrographs show representative Z-stack images acquired through confocal microscopy, with nuclear staining (DAPI) in blue, TSPO in green and various CNS cells of interest in red. TSPO co-localizing with the cellular markers of interest appears in yellow. Examples of co-localization areas are highlighted by the crosshair. **a** Examples of neuronal TSPO protein expression, as evaluated using the post-mitotic neuronal markers NeuN, SMI-32 and MAP-2. **b** Examples of non-neuronal TSPO protein expression, including expression in Iba1-positive microglia, GFAP-positive astrocytes and Glut1-positive vascular endothelial cells.

We further analyzed TSPO protein levels in neuronal and non-neuronal cells of the cornu ammonis by means of IF staining analyzed by CLSM, thereby using an antibody whose specificity was previously verified in a viable TSPO knockout mouse model [32]. In neurons, TSPO protein was found to co-localize with several post-mitotic neuronal markers, including neuronal nuclei (NeuN), neurofilament heavy polypeptide (SMI-32) and microtubule-associated protein 2 (MAP-2) (Fig. 2a) in hippocampal sections from adult (12 weeks old) C57BL6/N mice. The same CLSM analyses of IF staining also detected TSPO protein in non-neuronal cells, including microglia, astrocytes and vascular endothelial cells (Fig. 2b). Consistent with its mitochondrial localization, TSPO immunoreactivity generally appeared as defined punctae in both neuronal and non-neuronal cell types (Fig. 2). These data confirm that TSPO protein is present in both neuronal and non-neuronal cell populations under basal conditions.

### TSPO expression after selective neuronal activation

**Fig. 3:**
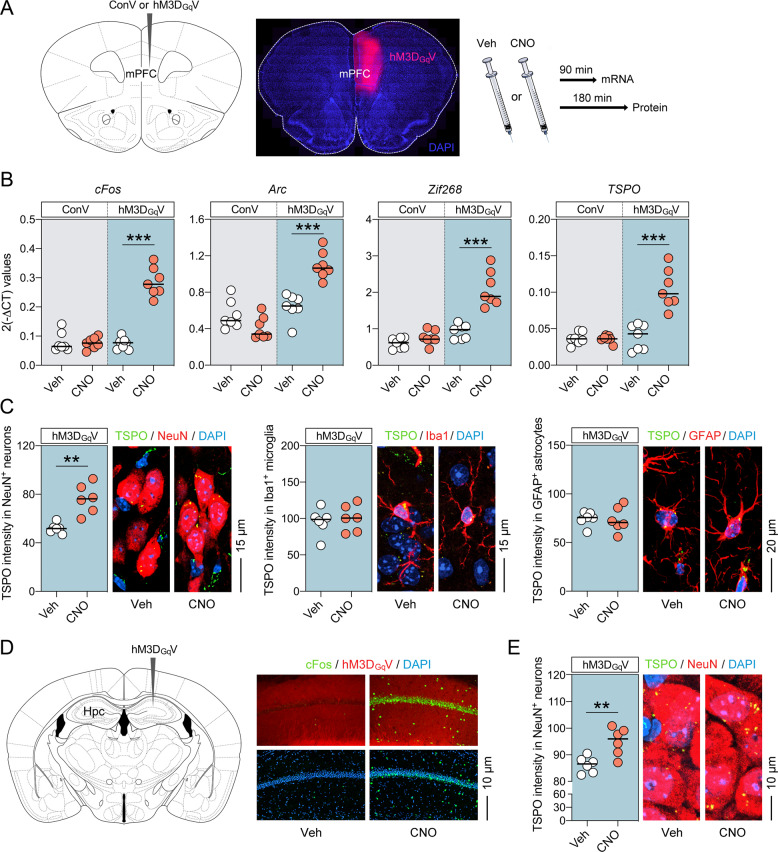
Increased TSPO levels following selective neuronal activation using the DREADD system in adult mice. **a** Schematic illustration and verification of the experimental approach. Mice were subjected to unilateral stereotactic injections of recombinant AAV expressing the modified human muscarinic M3 G-protein-coupled receptor under the control of the human synapsin-1 promoter (hM3D_Gq_V), or recombinant control AAV expressing a reporter gene under the same synapsin-1 promoter (ConV), into the medial prefrontal cortex (mPFC). Gene expression was assessed 90 min after vehicle (Veh) or clozapine-N-oxide (CNO, 1 mg/kg, i.p.) treatment, whereas protein expression was assessed 180 min post-treatment. The photomicrograph shows representative hM3D_Gq_V expression in the injected mPFC hemisphere. **b** mRNA levels of *cFos*, *Arc*, *Zif268* and *TSPO* (as measured by quantitative polymerase chain reaction) in the mPFC of ConV- or hM3D_Gq_V-injected mice 90 min after treatment with Veh or CNO. **c** Intensity (relative optical density) of TSPO protein co-localizing with NeuN-positive neurons (left), Iba1-positive microglia (middle) and GFAP-positive astrocytes in the mPFC of hM3D_Gq_V-injected mice 180 min after treatment with Veh or CNO (1 mg/kg, i.p.). The photomicrographs show representative images acquired through confocal microscopy, with nuclear staining (DAPI) in blue, TSPO in green and cell types of interest (neurons, microglia and astrocytes) in red. TSPO co-localizing with the cellular markers of interest appears in yellow. **d** Schematic illustration and immunohistochemical verification of unilateral, stereotactic injections of recombinant AAV expressing hM3D_Gq_V into the hippocampus (Hpc). The photomicrographs show representative confocal images of hM3D_Gq_V-injected mice at the level of the CA1 region of the Hpc, 180 min after treatment with Veh or CNO. Note the induction of cFos protein levels (in green) in CNO-treated relative to Veh-treated mice. **e** Intensity (relative optical density) of TSPO protein co-localizing with NeuN-positive neurons in the Hpc of hM3D_Gq_V-injected mice 3 h after treatment with Veh or CNO (1 mg/kg, i.p.). The photomicrographs show representative images acquired through confocal microscopy, with nuclear staining (DAPI) in blue, TSPO in green and NeuN in red. TSPO co-localizing with the cellular markers of interest appears in yellow. For all data, **P < 0.01 and ***P < 0.001; each dot in the scatter plot represents an individual animal.

We next examined whether TSPO mRNA and protein levels change in response to selective neuronal activation. To this aim, we first used a DREADD-based chemogenetic approach [28], in which we targeted mPFC neurons of adult mice via unilateral stereotactic injections of a recombinant AAV that expresses the modified human muscarinic M3 G-protein-coupled (Gq) receptor under the control of the human synapsin-1 promoter (hM3D_Gq_V) (Fig. 3a). This excitatory receptor was then activated by CNO (1 mg/kg, i.p.) [29, 30], leading to the selective activation of neurons. To control for possible off-target effects of CNO [33, 34], we compared CNO (relative to Veh) treatment in mice injected with hM3D_Gq_V and mice injected with a recombinant control AAV expressing a reporter gene under the same synapsin-1 promoter (ConV). As expected [28, 35], CNO treatment in hM3D_Gq_V-injected mice led to robust neuronal activation, as evident by the significant increase in the mRNA levels of several neuronal activity markers, including *cFos* (*t*_(12)_ = 4.90, *P* < 0.001), *Arc* (*t*_(12)_ = 4.63, *P* < 0.001) and *Zif268* (*t*_(12)_ = 4.35, *P* < 0.001) (Fig. 3b). CNO treatment in hM3D_Gq_V-injected mice similarly increased *TSPO* mRNA levels (*t*_(12)_ = 4.29, *P* < 0.001; Fig. 3b), suggesting that selective activation of mPFC neurons increases *TSPO* gene expression. Importantly, CNO treatment alone in ConV-injected mice had no effect on the mRNA levels of neuronal activity markers or *TSPO* (Fig. 3b). Hence, the CNO-mediated increase in *TSPO* mRNA expression, which was specifically observed in hM3D_Gq_V-injected mice, is unlikely to be accounted for by possible off-target effects of CNO, but rather arises from genuine DREADD-mediated neuronal activation.

We then used CLSM of IF-stained brain sections to explore whether DREADD-mediated neuronal activation also changes TSPO protein in the mPFC. The intensity of TSPO co-localizing with NeuN-positive neurons was significantly increased in the mPFC of hM3D_Gq_V-injected mice after CNO relative to Veh treatment (*t*_(10)_ = 3.65, *P* < 0.01; Fig. 3c). By contrast, CNO treatment did not alter the intensity of TSPO protein co-localizing with Iba1-positive microglia or GFAP-positive astrocytes. Consistent results were obtained in the Hpc of adult mice. Indeed, when applying the DREADD-based model of neuronal activation to the adult Hpc (Fig. 3d), a similar increase in TSPO protein in NeuN-positive Hpc neurons emerged after CNO treatment (relative to Veh treatment) in hM3D_Gq_V-injected mice (*t*_(10)_ = 3.59, *P* < 0.01; Fig. 3e). Taken together, these data demonstrate that enhancing neuronal activity in cortical (mPFC) or subcortical (Hpc) regions increases TSPO levels in neurons but not in glial cells.

### TSPO expression after neuronal activation under physiological and psychopharmacological conditions

**Fig. 4:**
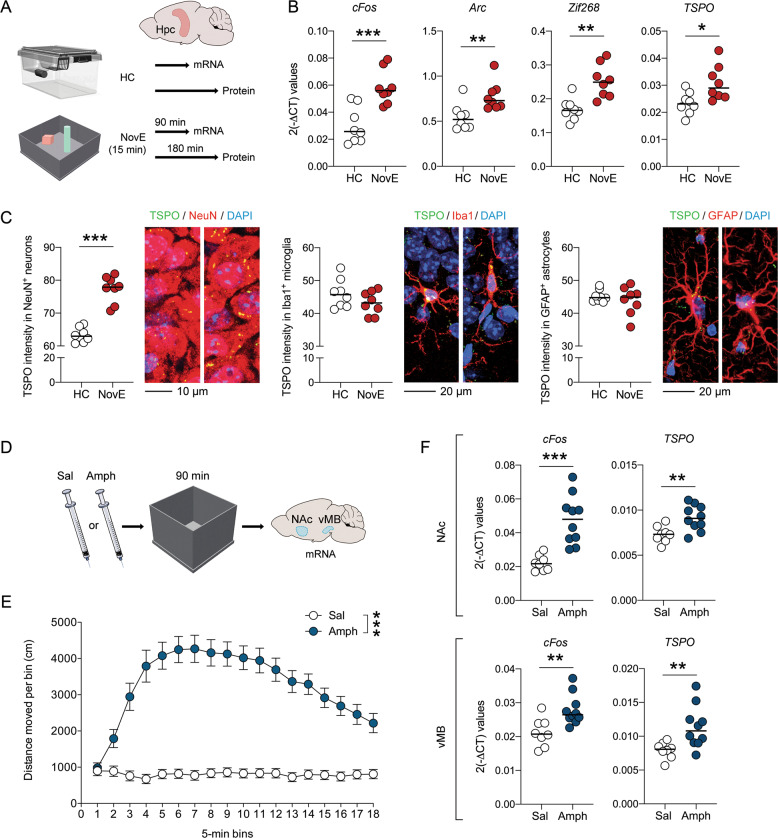
Increased TSPO levels following neuronal activation under physiological and psychopharmacological conditions in adult mice. **a** Schematic illustration of the experimental design used to assess TSPO levels after neuronal activation under physiological conditions. Mice were exposed to a novel environment (NovE) for 15 min. Gene expression was then assessed 90 min after NovE exposure, whereas protein expression was assessed 180 min post-exposure. Control mice were kept in their familiar home cage (HC) environment. **b** mRNA levels of *cFos*, *Arc*, *Zif268* and *TSPO* (as measured by quantitative polymerase chain reaction) in the hippocampus 90 min after NovE exposure, relative to mice kept in the HC environment. **c** Intensity (relative optical density) of TSPO protein co-localizing with NeuN-positive neurons (left), Iba1-positive microglia (middle) and GFAP-positive astrocytes in the Hpc 180 min after NovE exposure, relative to mice kept in the HC. The photomicrographs show representative images acquired through confocal microscopy, with nuclear staining (DAPI) in blue, TSPO in green and cell types of interest (neurons, microglia and astrocytes) in red. TSPO co-localizing with the cellular markers of interest appears in yellow. **d** Schematic illustration of the experimental design used to assess *TSPO* expression after neuronal activation under psychopharmacological conditions. Mice were injected with saline (Sal) or amphetamine (Amph, 2.5 mg/kg, i.p.) and then placed into an open field to verify Amph-induced hyperlocomotion. Gene expression was evaluated in the nucleus accumbens (NAc) and ventral midbrain (vMB) 90 min after Sal or Amph administration. **e** Distances moved after Sal or Amph administration as a function of 5-min bins. ***P < 0.001, based on repeated-measures ANOVA. **f** mRNA levels of *cFos* and *TSPO* (as measured by quantitative polymerase chain reaction) in the NAc and vMB 90 min after administration of Sal or Amph. *P < 0.05, **P < 0.01 and ***P < 0.001, based on two-tailed Student’s *t*-test; each dot in the scatter plot represents an individual animal.

We next explored whether TSPO expression also changes in response to neuronal activation induced by physiological and psychopharmacological manipulations. To assess the former, we compared neuronal activity markers as well as TSPO mRNA and protein levels in the Hpc of adult mice that were briefly (15 min) exposed to a novel environment (NovE), relative to mice that were kept in their familiar home cage (HC) environment (Fig. 4a). As expected [36], exposure to NovE stimulated neuronal activity in the Hpc, as evident by the significant increase in the mRNA levels of *cFos* (*t*_(14)_ = 4.48, *P* < 0.001), *Arc* (*t*_(14)_ = 3.12, *P* < 0.01) and *Zif268* (*t*_(14)_ = 2.99, *P* < 0.01) (Fig. 4b). Exposure to NovE also increased *TSPO* mRNA levels in the Hpc (*t*_(14)_ = 2.80, *P* < 0.05; Fig. 4b), showing that hippocampal *TSPO* gene expression parallels NovE-mediated activation of Hpc neurons. Subsequent CLSM analyses of IF-stained hippocampal sections confirmed this notion, showing that exposure to NovE increases the intensity of TSPO protein co-localizing with NeuN-positive neurons of the cornu ammonis (Fig. 4c). By contrast, exposure to NovE did not alter TSPO protein levels co-localizing with Iba1-positive microglia or GFAP-positive astrocytes in this brain area (Fig. 4c).

Finally, we investigated whether acute treatment with Amph, a psychostimulant drug that increases central dopamine and norepinephrine release [37], modulates the expression of TSPO. To this aim, we focused on the transcriptional activity of *TSPO* in two dopaminergic brain areas activated by Amph exposure, namely the NAc and vMB (Fig. 4d). As expected [31, 38], systemic Amph (2.5 mg/kg, i.p.) treatment enhanced locomotor activity (main effect of treatment in RM-ANOVA: *F*_(1,16)_ = 28.80, *P* < 0.001; Fig. 4e) and increased the mRNA levels of the neuronal activity marker, *cFos*, in both the NAc (*t*_(16)_ = 4.56, *P* < 0.001) and vMB (*t*_(16)_ = 3.27, *P* < 0.01) (Fig. 4f). Amph treatment also increased *TSPO* mRNA in both brain areas (NAc: *t*_(16)_ = 3.12, *P* < 0.01; vMB: *t*_(16)_ = 3.09, *P* < 0.01; Fig. 4f). Together, these findings demonstrate the capacity of physiologically or psychopharmacologically induced neuronal activation to upregulate *TSPO* mRNA and protein in the adult CNS.

## Discussion

The present study examined neuronal TSPO expression under basal conditions and after neuronal activation in several mouse models. In line with the qualitative findings from previous immunohistochemical studies [4, 14, 15], scRNA-seq analysis and CLSM of IF-stained brain sections identified relatively low but clearly detectable TSPO mRNA and protein in neurons under basal conditions. To our knowledge, the present scRNA-seq analysis of *TSPO* mRNA is the first to take into account the entire spectrum of hippocampal cells in adult mice. This analysis provided an unbiased approach of comparing basal *TSPO* gene expression in different hippocampal cell populations, and as such, it corroborates the existing evidence of the multi-cellular expression profile of *TSPO* [4, 14, 15, 39, 40]. This non-selective cellular expression pattern is not unprecedented, given that TSPO is a mitochondrial protein with various plausible functions pertaining to a number of CNS cell types [16, 17, 32, 41].

We further identified neuronal activation to represent a novel non-inflammatory mechanism by which TSPO levels can be altered in the brain. Indeed, we found consistent increases in TSPO gene expression and protein levels after neuronal activation using a spectrum of stimuli, including selective stimulation (DREADD system), physiological activation (NovE exposure) and psychopharmacological manipulation (Amph treatment). These data provide the first direct evidence for a causal relationship between neuronal activity and TSPO levels in the CNS. The fact that these effects involved increases in *TSPO* mRNA further implies de-novo synthesis of TSPO in response to neuronal stimulation. Moreover, neuronal activation upregulated TSPO selectively in neurons, but not in in glial cells, pointing toward specific neuronal effects.

On speculative grounds, neuronal activity may lead to higher TSPO levels in neurons because of increased energy demands. Circumstantial evidence for this hypothesis stems from previous findings implicating TSPO in mitochondrial bioenergetics and ATP production [16, 17]. This hypothesis is also consistent with our scRNA-seq analysis, demonstrating that basal *TSPO* expression in neurons is highest in those neuronal sub-populations that are characterized by transcriptional indices of increased protein synthesis and oxidative phosphorylation. More specifically, we found higher *TSPO* mRNA levels in a neuronal sub-population showing increased expression of genes involved in EIF2 signaling pathway, which in turn is essential for initiating the translation and protein synthesis in neurons [42]. Consistent with the role of TSPO in mitochondrial bioenergetics [16, 17], this neuronal sub-cluster also showed increased expression of genes involved in oxidative phosphorylation. Hence, the extent to which neuronal *TSPO* is expressed under basal conditions appears to parallel the transcriptional activity of neurons, which in turn may raise the cellular demand in oxidative phosphorylation and subsequent energy production.

This hypothesis may also explain why under basal conditions *TSPO* mRNA is generally more abundant in ependymal cells, vascular endothelial cells and microglia than in neurons. Indeed, these non-neuronal cell types are characterized by marked energy demands, even under non-pathological or homeostatic conditions. For example, ependymal cells are a specialized type of epithelial cells that line the ventricular system of the brain and play a key role in the homeostasis of CSF [43]. They possess a large number of mitochondria, which are required to meet the energy demand of ependymal cells for CSF transport and other homeostatic processes [44]. Therefore, it appears logically consistent that the sqRNA-seq analysis identified ependymal cells to be the cell population with the most abundant *TSPO* transcripts. Likewise, microglia are now known to be “never-resting cells” that constantly survey the CNS for invading pathogens, changes in the physiological microenvironment and/or CNS injury [45, 46]. The surveying activities of microglia arguably require energy, which may be readily mirrored by the abundance of *TSPO* transcripts, even under non-pathological, homeostatic conditions. Upon activation by pathological insults, microglia rapidly alter their morphological appearance and transcriptional profiles, increase their motility and phagocytic activity and produce and secrete various factors that are integral for combating pathogens and/or initiating and promoting tissue remodeling and repair. All of these responses require additional energy and corresponding adaptations in mitochondrial bioenergetics [47, 48], such that the upregulation of TSPO in microglia under pathological conditions [4–6] may generally reflect the cells’ increased demand in energy production. If confirmed by future investigations, the upregulation of neuronal TSPO in response to neuronal activation may reflect similar bioenergetic processes.

It is currently unknown whether altered neuronal activity may contribute to changes in TSPO binding as observed in PET studies of neurological and psychiatric disorders. Several recent findings suggest, however, that this possibility may indeed exist. In a cohort of long-term cannabis users and control subjects, for example, Da Silva et al. [49] found a positive correlation between TSPO levels and states of stress and anxiety, both of which have stimulatory effects on limbic neurons [50]. Combining TSPO PET imaging with proton magnetic resonance spectroscopy further revealed a positive association between TSPO binding and the levels of γ-aminobutyric acid (GABA) in the mPFC of people at clinical high risk for psychosis and healthy volunteers [51]. Even though a direct neuronal contribution to altered TSPO expression or binding in psychosis still awaits verification, these findings are in line with the notion that glial cells are unlikely to be the sole cellular sources underlying these changes [4, 12, 52]. In fact, given our findings presented here, the prefrontal downregulation of TSPO occurring in a subset of patients with psychotic disorders [12] and in disease-relevant animal models [53] may, at least in part, reflect reduced neuronal activity in PFC structures or “hypofrontality”. Finally, it has recently been found that the variable cortical burden of TSPO does not correlate with the burden of activated microglia or reactive astrocytes in the brains of late-stage Alzheimer’s disease patients [54]. Future studies will be necessary to determine whether loss of neurons and/or blunted neuronal activity may contribute to reduced TSPO expression occurring in a substantial portion of patients with late-stage Alzheimer’s disease or Lewy body dementia [54].

We appreciate that our study has a number of limitations. First, because of ethical and technical reasons, we investigated the relationship between neuronal activity and TSPO levels in mouse models only. Therefore, we cannot rule out possible species differences, which are relevant in the context of inflammatory regulation of TSPO expression in microglia and macrophages [55]. Second, we included mice of one sex (males) and age (early adulthood) only. In view of the recently identified influence of sex [56] and age [56, 57] on brain TSPO expression, additional studies are warranted to explore whether our findings are generalizable across sex and different ages.

Despite these limitations, we conclude that our findings have important implications for implementing and interpreting TSPO-based biomarker studies. TSPO is commonly used as a biomarker of neuroinflammation in general, and of microglial activation in particular [9–11]. However while TSPO-based PET imaging signals have often been considered to support neuroinflammatory theories of disease [9–11], it appears difficult, if not impossible, to define the precise pathophysiological meanings and cellular sources of altered TSPO binding in such studies. In some pathological conditions, increased TSPO expression may primarily reflect ongoing inflammatory processes [58–60], whereas in others it may signify anomalies in cellular metabolism and energy production [17], oxidative stress [61], and/or neuronal activity (present findings). The ambiguity of conceiving TSPO simply as a biomarker of neuroinflammation or microglial activation warrants caution, and supports the urgent development of novel approaches for assessing changes in inflammatory states in the brain [4, 62, 63].

## Supplementary Information

Supplementary Information
