# Supplementary material for: Neuronal activity increases translocator protein (TSPO) levels
Source: Mol Psychiatry. 2020 May 12;26(6):2025–37. doi: 10.1038/s41380-020-0745-1 (PMC8440208; doi:10.1038/s41380-020-0745-1)
Supplement: Supplementary file 1 — Supplementary Information [file 41380_2020_745_MOESM1_ESM.pdf]

SUPPLEMENTARY INFORMATION

Neuronal Activity Increases Translocator Protein (TSPO) Levels

Tina Notter[1,*], Sina M. Schalbetter[2], Nicholas E. Clifton[1,3], Daniele Mattei[2], Juliet Richetto[2], Kerrie Thomas[1], Urs Meyer[2,4,§], Jeremy Hall[1,3,§]

[1]Neuroscience and Mental Health Research Institute, Cardiff University, Cardiff, Wales, UK.
[2]Institute of Pharmacology and Toxicology, University of Zurich-Vetsuisse, Zurich, Switzerland.
[3]MRC Centre for Neuropsychiatric Genetics and Genomics, Division of Psychological Medicine and Clinical Neurosciences, Cardiff University, Cardiff, UK.
[4]Neuroscience Centre Zurich, University of Zurich and ETH Zurich, Zurich, Switzerland.

**Correspondence:* Tina Notter, Ph.D., Neuroscience and Mental Health Research Institute, Cardiff University, Hadyn Ellis Building, Maindy Road, Cardiff, CF24 4HQ, Wales, UK.
Email: nottert@cardiff.ac.uk

[§]Shared senior authorship

SUPPLEMENTARY METHODS

Brain Dissociation and Cell Isolation for Single-Cell RNA Sequencing

The protocol has been adapted from our previous protocol[1] and from the procedures provided by Miltenyi (adult brain dissociation kit, ABDK, Miltenyi). It was further optimized for the dissociation and isolation of cells from brain tissue with small volumes such as mouse hippocampi.

The animals were deeply anesthetized with an overdose of Nembutal (Abbott Laboratories, North Chicago, IL, USA) and transcardially perfused with 15 mL ice-cold, calcium- and magnesium-free Dulbecco's phosphate-buffered saline (DPBS; pH 7.3-7.4; Thermo Fisher Scientific; Zurich Switzerland) via a 20 mL syringe and a 23 G needle (25 mm length). The brains were quickly removed and washed with ice-cold DPBS, after which the hippocampi were dissected on a cooled petri dish and placed in ice-cold Hibernate-A medium.

Mechanical dissociation (MD) at 4°C was carried out on ice, while all the solutions were kept at 4°C. The hippocampi were dissociated in 1.5 mL Hibernate-A medium (Brainbits, Springfield, IL, USA) in a 1 mL Dounce homogenizer with a loose pestle (Active Motif, La Hulpe, Belgium). The tissue was gently dounced until no tissue pieces were visible. The homogenized tissue was then sieved through a 70 µm cell strainer mounted onto a 50 mL Falcon tube, after which the Dounce homogenizer was washed twice with 1 mL Hibernate-A, whereby each wash was poured onto the cell strainer. The homogenized hippocampi were then transferred to 5 mL Eppendorf tubes and kept on ice as described above.

After MD, the homogenates were pelleted at 450xg for 6 minutes at 4°C in a swing-bucket rotor centrifuge (Eppendorf, Schönenbuch, Switzerland). The supernatants were removed and 1 mL ice-cold DPBS (pH 7.3-7.4) was added to all samples. The pellets were then re-suspended with a P1000 micropipette. After re-suspension, the final volume in each tube was brought to 1.5 mL. 500 µL of freshly prepared isotonic Percoll solution (Merck, Zug, Switzerland) was added to each sample (final volume: 2 mL) and mixed well (applying a pipette-tip cut-off to optimize the mixing). Percoll was rendered isotonic by mixing 1 part of 10x calcium- and magnesium-free DPBS (pH 7.3-7.4) with 9 parts of Percoll. Importantly, the pH of Percoll was adjusted to 7.3-7.4 with 5 molar hydrochloric acid before starting the isolation procedure. The Percoll solution was mixed properly with the cell suspension, after which 2 mL of DPBS were gently layered on top of it with a pipette boy set on the slowest speed, creating two separate layers. The samples were centrifuged for 10 minutes at 3000 x

g. The centrifugation resulted in an upper layer consisting of DPBS and a lower layer consisting of Percoll. The 2 layers were separated by a disk of myelin and debris, while the cells were located at the bottom of the tube. The layers were aspirated, leaving about 500 μL of solution on top of the pellet as some cells, depending on their size, can float in Percoll right above the pellet. The cells were then washed once in DPBS by gently adding 4 mL DPBS, closing the tube and gently tilting it in order to mix the remaining Percoll with the added DPBS. The cells were then pelleted once more by centrifuging them at 460 x g for 10 minutes at 4°C before further processing for single-cell RNA sequencing (as described in the *Main Manuscript*).

Stereotaxic Surgery

The mice were anesthetized by inhalation of 2.5-3% isoflurane (ZDG9623V, Baxter, Switzerland) in oxygen. Once fully anesthetized, they were injected with the analgesic Temgesic (buprenorphine, 0.1 mg/kg, s.c., Reckitt Benckiser, Switzerland) and placed in to the stereotaxic frame (MTM-3, World Precision Instruments, USA) whilst kept under constant isoflurane/oxygen flow (1-3% isoflurane in 200 mL/min oxygen). During the entire surgery mice were kept on a heat map (ATC1000, World Precision Instruments, USA) to control their body temperature. The mice received a unilateral injection of 600 nL of AAV at a flow rate of 10 nL/s into either the medial prefrontal cortex (mPFC) (anteroposterior (AP) = +2 mm, mediolateral (ML) = +0.3 mm, dorsoventral (DV) = −2.0 mm, with reference to bregma) or the dorsal hippocampus (Hpc) (AP = −2, ML = +1.6, DV = −1.9, with reference to bregma) using a Nanofil needle and syringe (NANOFIL, NF35BV, World Precision Instruments, USA) connected to a automated pump system (UMP3T-1, World Precision Instruments, USA). After injection of the AAV, the needle was kept in place for 5 minutes to avoid reflux of the virus and then retracted. The incision was sutured with a surgical thread (G0932078, B. Braun, Switzerland). For recovery the mice were initially placed into a temperature-controlled chamber (Harvard Apparatus, USA), after which they were placed back into their home cage and closely monitored for 3 consecutive days.

Microscopy and Immunohistochemical Data Analysis

Data collection was performed with confocal laser scanning microscopy (LSM-700; Zeiss, Jena, Germany) using a 40× (oil, NA 1.4) objective with a zoom of 2 (for neurons) and 1.5 (for microglia and astrocytes) using sequential acquisition of separate wavelength channels to avoid fluorescence crosstalk. Laser intensities for each separate channel were set and kept constant during the entire

image acquisition. For each animal, 12 images were randomly acquired from 4 consecutive sections containing the prelimbic and infralimbic areas of the mPFC (bregma: +2.0 to +1.5 mm), whereas 36 images were acquired from 8 consecutive sections containing the cornu ammonis (CA) region of the Hpc (bregma −1.5 to −3.5 mm).

TSPO intensities (mean grey value) co-localized with NeuN-positive neurons, Iba1-positive microglia or GFAP-positive astrocytes were measured and calculated on single plane images using a custom-made macro (kindly provided by Prof. Jean-Marc Fritschy, Institute of Pharmacology and Toxicology, University of Zurich, Switzerland) developed for the ImageJ software. This macro has previously been validated for immunohistochemical co-localization studies[2]. In brief, Gaussian filter, background subtraction and a threshold (moments dark) were applied to the images for each channel. The settings for each marker were adjusted so that an optimal representation of TSPO, neurons, microglia and astrocytes was achieved and kept constant during image analyses. The adjusted settings for TSPO, NeuN, Iba1 and GFAP served to define the co-localization. Co-localization was defined as pixel clusters in the TSPO channel that overlapped with pixel clusters in the NeuN, Iba1 or GFAP channels, with a set size cut-off at 0.1 μm^2. Pixel brightness (mean grey value) of co-localized TSPO (TSPO-positive pixel clusters defined as co-localized in the adjusted images) was measured and calculated from the raw data set. Co-localized TSPO intensity was averaged over the acquired images (12 images in the mPFC and 36 images in the CA of the Hpc) per animal.

Experiment	Number of Animals
DREADD (mPFC)<br>qPCR	7 (ConV – Veh)<br>7 (ConV – CNO)<br>7 (hM3D$_{Gq}$V – Veh)<br>7 (hM3D$_{Gq}$V – CNO)
DREADD (mPFC)<br>IHC	6 (hM3D$_{Gq}$V – Veh)<br>6 (hM3D$_{Gq}$V – CNO)
DREADD (Hpc)<br>IHC	6 (hM3D$_{Gq}$V – Veh)<br>6 (hM3D$_{Gq}$V – CNO)
Novel Environment<br>qPCR	8 (HC)<br>8 (NovE)
Novel Environment<br>IHC	8 (HC)<br>8 (NovE)
Apmphetamine	8 (Sal)<br>10 (Amph)

Supplementary Table S1. Number of animals (male C57BL6/N mice; 12 weeks old) in each experimental condition and series of experiment used in the study.

Gene	Forward Primer	Reverse Primer	Probe
Arc	5’-TGCAGATTGGTAAGTTGCCGA-3’	5’-TGTGCAACCCTTTCAGCTCT-3’	5’-TTCTGTTGACCGAAGTGTCCA-3’
cFos	5’-TCCTTACGGACTCCCCAC-3’	5’-CTCCGTTTCTCTTCCTCTTCAG-3’	5’-TGCTCTACTTTGCCCCTTCTGCC-3’
Zif-268	5’- AGCGCCTTCAATCCTCAAG-3’	5’- TTTGGCTGGGATAACTCGTC-3’	5’- CAACCCTATGAGCACCTGACCACA-3’
36B4	5’-AGATGCAGCAGATCCGCAT-3’	5’-GTTCTTGCCCATCAGCACC-3’	5’-CGCTCCGAGGGAAGGCCG-3’

Supplementary Table S2. List of custom-designed probe and primer sequences used for the various genes of interest and reference gene (*36B4*). They were purchased from Eurofins Genomics GmbH (Germany).

Target	Distributor	Description, Cat#	Dilution
Translocator protein (TSPO)	Abcam, Cambridge, UK	Rabbit polyclonal, Ab109497	1:6’000
Ionized calcium-binding adaptor molecule 1 (Iba1)	EMD Millipore, Billerica, USA	Mouse monoclonal, MABN92	1:1’000
Ionized calcium-binding adaptor molecule 1 (Iba1)	Abcam, Cambridge, UK	Goat polyclonal, Ab5076	1:1’000
Glial fibrillary acidic protein (GFAP)	EMD Millipore, Billerica, USA	Mouse monoclonal, MAB360	1:5’000
Glucose transporter 1 (Glut1)	Abcam, Cambridge, UK	Mouse monoclonal, Ab40084	1:2’000
Neuronal nuclei (NeuN)	Synaptic Systems, Germany	Guinea pig polyclonal, 266 004	1:1’000
Anti-neurofilament H (NF-H), nonphosphorylated (SMI-32)	Lucerna-Chem, Switzerland	Mouse monoclonal, SMI-32R	1:2’000
Microtubule-associated protein 2 (MAP-2)	Sigma-Aldrich, Switzerland	Mouse monoclonal, M-1406	1:4’000

Supplementary Table S3. List of primary antibodies used in this study. The specificity and efficacy of the primary antibody against TSPO have been validated in mice before, including mice with a complete genetic deficiency of the *TSPO* gene[3] Antibodies against Iba1 and GFAP were used to used to detect microglia and astrocytes, respectively, whereas primary antibody against Glut1 was used to visualize endothelial cells of the brain vasculature according to protocols established and validated before[4, 5]. The selected antibodies against Iba1, GFAP and Glut1 have been validated thoroughly in previous studies using C57BL6/N mice[2, 5]. Antibodies against NeuN, SMI-32 and MAP-2 were used to detect and visualize post-mitotic neurons.

SUPPLEMENTARY RESULTS

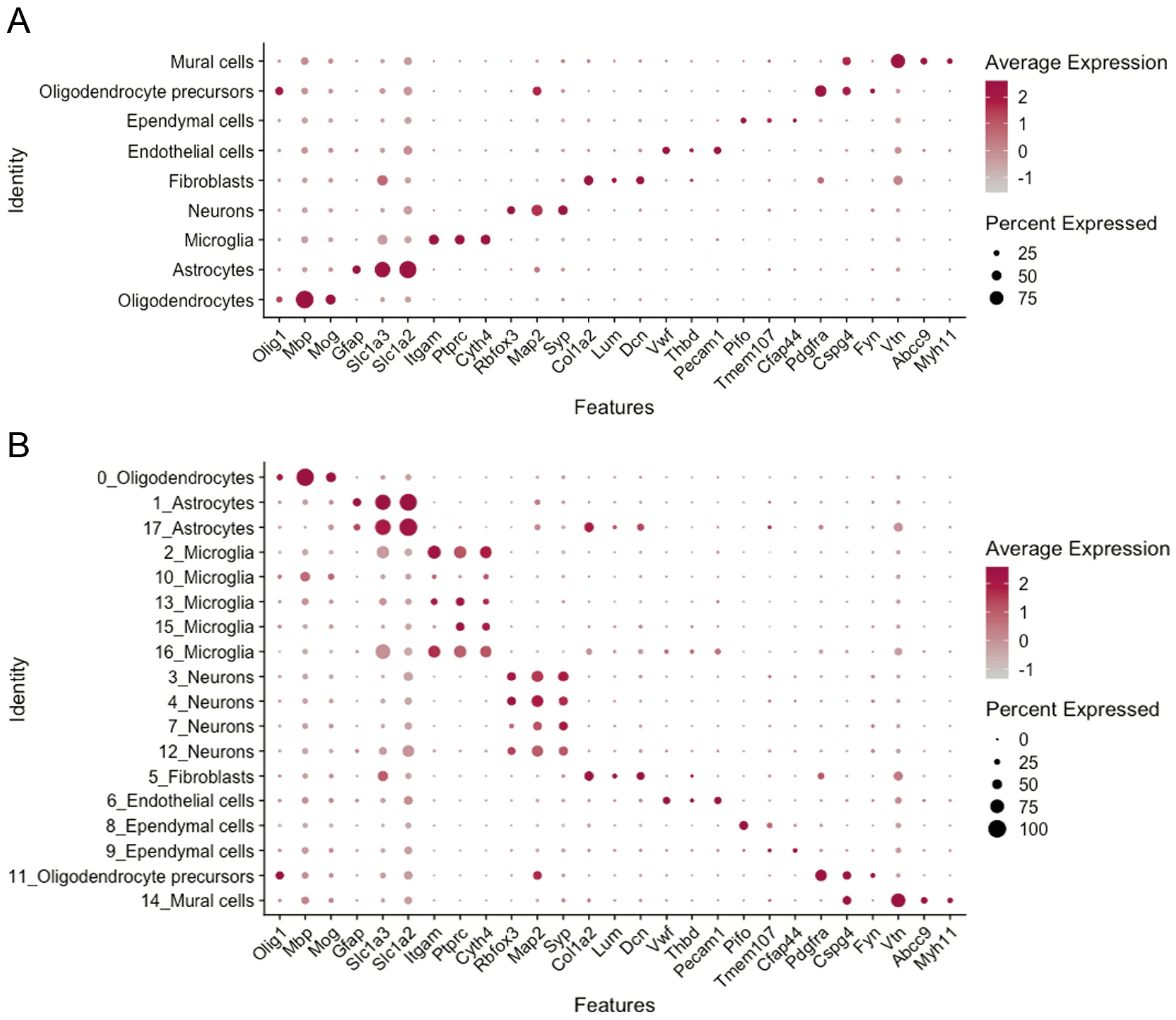

Supplementary Figure S1 Gene-sets used for cell-type identification. **(A)** The figure shows the expression levels of select genes and the percentage of cells in each of the 9 main cell population clusters. **(B)** The figure shows the expression levels of select genes and the percentage of cells in each of the 18 cell clusters containing distinct cell sub-populations. Gene-set enrichments to identify each cell type were acquired from previously published murine hippocampal single cell RNA-sequencing studies[6, 7].

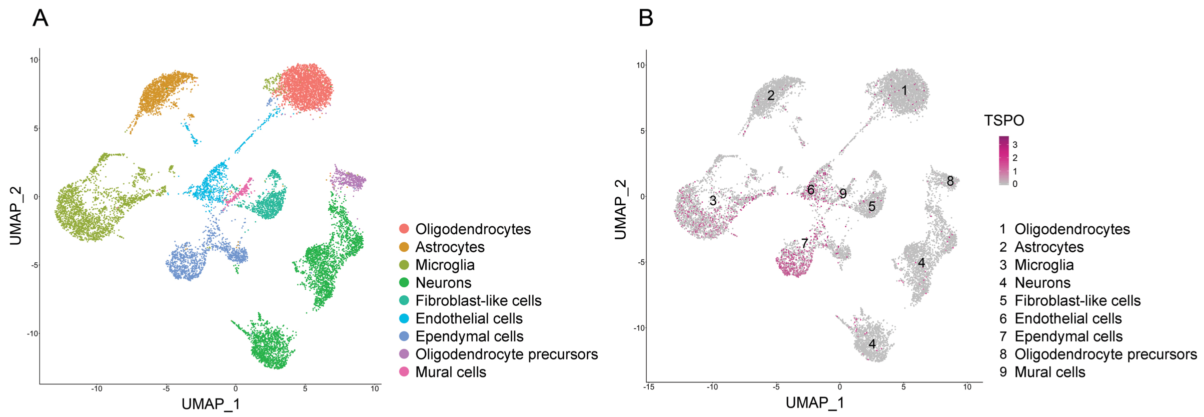

***Supplementary Figure S2*. (A)** Visualization of single-cell RNA-sequencing (scRNA-seq) data uniform manifold approximation and projection (UMAP) scores, showing the clustering of 9 main cell populations in the hippocampus of adult (12 weeks old) mice (C57BL6/N). Corresponding t-distributed stochastic neighbor embedding (tSNE) scores are shown in *Figure 1 (Main Text).* **(B)** *TSPO* expression (in purple) in individual clusters of cells as detected by scRNA-seq.

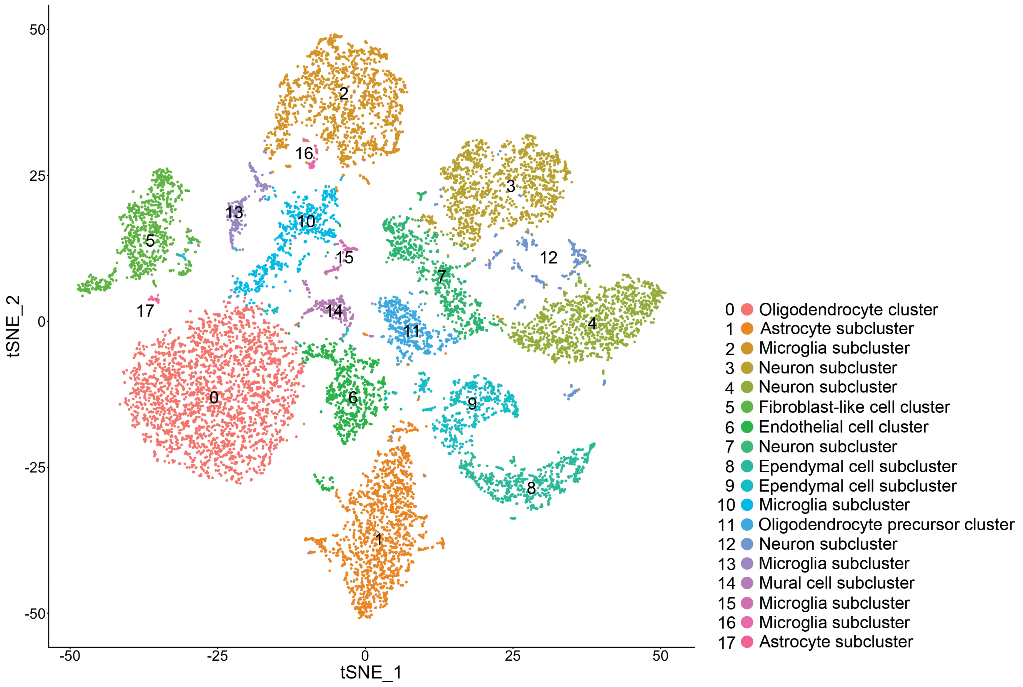

***Supplementary Figure S3*.** Visualization of single-cell RNA-sequencing (scRNA-seq) data using t-distributed stochastic neighbor embedding (tSNE), showing the clustering of 18 cell clusters containing distinct cell sub-populations in the hippocampus of adult (12 weeks old) mice (C57BL6/N).

SUPPLEMENTARY REFERENCES

1. Mattei D, Ivanov A, Ferrai C, Jordan P, Guneykaya D, Buonfiglioli A *et al.* Maternal immune activation results in complex microglial transcriptome signature in the adult offspring that is reversed by minocycline treatment. *Translational psychiatry* 2017; **7**(5)**:** e1120.

2. Notter T, Coughlin JM, Gschwind T, Weber-Stadlbauer U, Wang Y, Kassiou M *et al.* Translational evaluation of translocator protein as a marker of neuroinflammation in schizophrenia. *Molecular psychiatry* 2018; **23**(2)**:** 323-334.

3. Banati RB, Middleton RJ, Chan R, Hatty CR, Kam WW, Quin C *et al.* Positron emission tomography and functional characterization of a complete PBR/TSPO knockout. *Nature communications* 2014; **5:** 5452.

4. Choeiri C, Staines W, Messier C. Immunohistochemical localization and quantification of glucose transporters in the mouse brain. *Neuroscience* 2002; **111**(1)**:** 19-34.

5. Giovanoli S, Engler H, Engler A, Richetto J, Voget M, Willi R *et al.* Stress in puberty unmasks latent neuropathological consequences of prenatal immune activation in mice. *Science (New York, NY)* 2013; **339**(6123)**:** 1095-1099.

6. Hamilton DJ, White CM, Rees CL, Wheeler DW, Ascoli GA. Molecular fingerprinting of principal neurons in the rodent hippocampus: A neuroinformatics approach. *Journal of pharmaceutical and biomedical analysis* 2017; **144:** 269-278.

7. Saunders A, Macosko EZ, Wysoker A, Goldman M, Krienen FM, de Rivera H *et al.* Molecular Diversity and Specializations among the Cells of the Adult Mouse Brain. *Cell* 2018; **174**(4)**:** 1015-1030.e1016.
